# Time-Resolved Tracking of Mutations Reveals Diverse Allele Dynamics during *Escherichia coli* Antimicrobial Adaptive Evolution to Single Drugs and Drug Pairs

**DOI:** 10.3389/fmicb.2017.00893

**Published:** 2017-05-24

**Authors:** Rachel A. Hickman, Christian Munck, Morten O. A. Sommer

**Affiliations:** Bacterial Synthetic Biology, Novo Nordisk Foundation, Center for Biosustainability, Technical University of DenmarkKongens Lyngby, Denmark

**Keywords:** antibiotics, allelic dynamics, population frequency sequencing

## Abstract

Understanding the evolutionary processes that lead to antibiotic resistance can help to achieve better treatment strategies. Yet, little is known about the dynamics of the resistance alleles during adaptation. Here, we use population sequencing to monitor genetic changes in putative resistance loci at several time-points during adaptive evolution experiments involving five different antibiotic conditions. We monitor the mutational spectra in lineages evolved to be resistant to single antibiotics [amikacin (AMK), chloramphenicol (CHL), and ciprofloxacin (CIP)], as well as antibiotic combinations (AMK + CHL and CHL + CIP). We find that lineages evolved to antibiotic combinations exhibit different resistance allele dynamics compared with those of single-drug evolved lineages, especially for a drug pair with reciprocal collateral sensitivity. During adaptation, we observed interfering, superimposing and fixation allele dynamics. To further understand the selective forces driving specific allele dynamics, a subset of mutations were introduced into the ancestral wild type enabling differentiation between clonal interference and negative epistasis.

## Introduction

Bacteria have an impressive ability to adapt to changes in their environment. Their large population sizes and short generation times allow bacteria to rapidly evolve and adapt in response to environmental perturbations. This either occurs via the horizontal acquisition of new genes or through mutations of existing genes. In the latter case, random single nucleotide variants (SNVs) or insertions/deletions (INDELs) in the genome drive the evolution of new traits.

The ability of a mutated allele to establish itself within a population depends on complex interplay between the positive selection for the conferred benefit of the mutation, random genomic drift, and negative selection against any associated fitness costs of the mutation. Consequently, novel mutant alleles often arise within a bacterial population but whilst they are at a low frequency they can easily be lost due to genomic drift (Gerrish and Lenski, 1998). If a novel mutant allele provides an advantageous phenotype, then it can become established within the population (Fogle et al., 2008). Once a mutant allele becomes established within a bacterial population, it can become fixed or compete with other subsequent mutant alleles that are present within the population; this process is known as clonal interference (Gerrish and Lenski, 1998; Fogle et al., 2008). The population size is often the governing factor that determines whether a mutant allele becomes fixed or lost within a bacterial population. In a small bacterial population, the establishment of mutations is a rare event; thus, the mutant allele has a greater likelihood of becoming fixed. By contrast, the opposite is true in large bacterial populations in which competition between mutations is more likely to occur, thus reducing the likelihood of a mutation becoming fixed within a population (Wilke, 2004; Good et al., 2012).

By identifying SNVs and INDELs at the population level at different time points during adaptation to a physical or a chemical perturbation, it is possible to uncover the trajectories through which a population evolves. Studies of such evolutionary trajectories have identified the population genetic dynamics underlying adaptation to different diverse perturbations, such as glucose being a limiting nutrient (Barrick et al., 2009; LaCroix et al., 2015) and antibiotic exposure (Toprak et al., 2011; Zhang et al., 2015; Feng et al., 2016).

An interesting phenomenon that often accompanies mutational responses to an environmental perturbation is the emergence of collateral effects. This phenomenon describes situations in which the adaptive mutations acquired in response to one perturbation affect the cell’s tolerance of other perturbations. Such collateral effects have been found in bacteria (Szybalski and Bryson, 1952; Imamovic and Sommer, 2013; Lázár et al., 2013; Munck et al., 2014; Oz et al., 2014; Pál et al., 2015), viruses (Miedema et al., 2013), and human cell lines (Cerezo et al., 2015; Zhao et al., 2016).

Fundamentally, collateral effects can be divided into two categories: collateral resistance and collateral sensitivity. Collateral resistance involves situations in which increased tolerance to one xenobiotic perturbation also provides increased tolerance to other xenobiotic perturbations. By contrast, collateral sensitivity describes situations in which increased tolerance to one perturbation is accompanied by increased sensitivity to other perturbations. In a recent study, we investigated how bacterial populations respond to a dual selection pressure relative to a single selection pressure (Munck et al., 2014). Over the course of 14 days, bacterial populations adaptively evolved to either single antibiotics or combinations of two antibiotics. Interestingly, we found that the presence of collateral sensitivity between two drugs significantly limited the population’s capacity to evolve resistance. In the current study, we follow the evolutionary events at key genetic loci in bacterial populations that have been exposed to increasing concentrations of either a single antibiotic or combinations of two antibiotics (Munck et al., 2014). We investigated lineages that evolved resistance to amikacin (AMK), chloramphenicol (CHL), ciprofloxacin (CIP), amikacin + chloramphenicol (AMK–CHL) and chloramphenicol + ciprofloxacin (CHL–CIP). The AMK–CHL drug combination was selected because it was found to generate evolutionary tension that suppressed the evolution of antimicrobial resistance, whereas the CHL–CIP combination was chosen because the component drugs have complementary evolutionary trajectories that enhance resistance evolution toward the combination. In this study, amplicon population sequencing and genome engineering were used to characterize the evolutionary dynamics of the responses to the individual antibiotics and antibiotic combinations.

## Materials and Methods

### Bacterial Strains

For the adaptation evolution experiment and the generation of samples for collection in this study, the bacterial strain used was MG1655, which is an *E. coli* K-12 MG1655 wild type (Blattner et al., 1997). Further characterization experiments were performed on successfully recombined strains to reintroduce given mutations (**Table 1**).

**Table 1:** Recombineered bacterial strains.

Bacterial strain	Observed bacterial population variant	Amino acid change	Genomic change
MG1655: *marR* C328T	CHL Lineage C	*marR* Gln110^∗^	*marR* C328T
MG1655: *marR* T251A	CHL–CIP Lineage A	*marR* Val84Glu	*marR* T251A
MG1655: *gyrA* 247_249delTCG	CHL–CIP Lineage A	*gyrA* Ser83del	*gyrA* 247_249delTCG
MG1655: *rob* G467A	CHL Lineage C	*rob* Arg156His	*rob* G467A
MG1655: *gyrA*247_249delTCG + *marR* T251A	CHL–CIP Lineage A	*gyrA* Ser83del + *marR* Val84Glu	*gyrA* 247_249delTCG + *marR* T251A
MG1655: *marR* C328T + *rob* G467A		*marR* Gln110^∗^ + *rob* Arg156His	*marR* C328T + *rob* G467A
MG1655	Ancestral wild type		

*^∗^Non-sense mutation.*

### Sample Revival and DNA Extraction

Microtiter plates saved from Munck et al. (2014) were defrosted for 30 min at 4°C. To prevent aerosol contamination, the plates were centrifuged at 300 ×*g* for 5 s before seal removal. To revive our samples, 150 μL were transferred to new tubes with 500 μL LB media and incubated at 37°C for 3 h. Genomic DNA was extracted using Genomic Mini Kits in accordance with the manufacturer’s protocol (A&A Biotechnologies, Gdynia, Poland).

### Resistance Allele Primer Design

**FIGURE 1:**
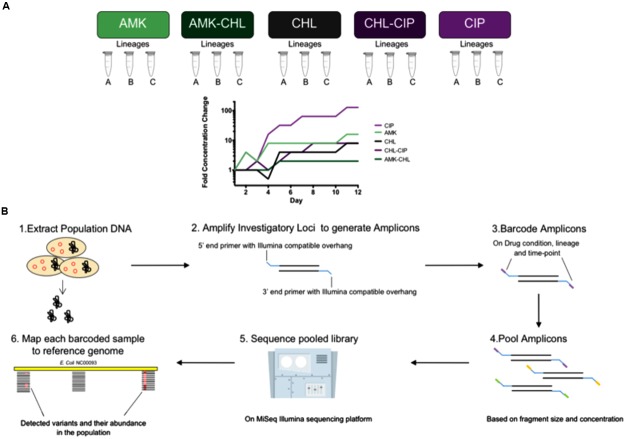
**Experimental overview of the investigation from sample collection description, adaptation laboratory evolution (ALE) and amplicon frequency workflow. (A)** Background for our frozen sample conditions showing five drug conditions (AMK, CHL, CIP, AMK–CHL, and CHL–CIP) each with three lineages (Lineages A, B, C) covering six time points (days 2, 4, 6, 8, 10, and 12) for the laboratory adaptation experiments. **(B)** The workflow used to assess different loci from different drug conditions, lineages, and time points in which (1) the DNA was extracted from the different samples, (2) each sample has amplicons for each locus generated by primers that had a DNA amplifying region and an Illumina Nextera XT compatible overhang, (3) these were then barcoded using indices from the Illumina Nextera XT library preparation kit, (4) all of the generated amplicons were pooled together based on fragment size and concentration, (5) the pooled amplicon library was sequenced on the MiSeq Illumina sequencing platform, (6) each sample was sorted by barcode, and each was used to generate a file to map to the reference genome and call variants in the bacterial population and to calculate their frequencies.

The loci that were selected for sequencing were chosen based on knowledge of their involvement in antibiotic resistance and their mutation profiles in the end-point sequenced original evolved lineages, and the population level loci sequencing was performed on days 2, 4, 6, 8, 10 and 12 (**Figure 1A**). For this study, we monitored the following mutations of AMK: *cpxA*, *fusA*, and *sbmA*; for CHL, we monitored the following mutations: *acrR*, *marR*, *rob*, and *soxR*; for CIP, we monitored the following mutations: *acrR*, *gyrA*, and *soxR*; for AMK + CHL, we monitored the following mutations: *acrR*, *cpxA*, *fusA*, *marR*, *rob*, and *soxR*; and for CHL + CIP, we monitored the following mutations: *acrR*, *gyrA*, *marR*, *rob*, and *soxR*. We believe that these loci cover important genomic locations that could begin to unravel important interactions in intrinsic antibiotic resistance. The primers were designed with overhangs to allow for barcoding with Nextera XT indices in accordance with the Illumina 16S Metagenomic Sequencing Protocol (Illumina Protocol Online) to amplify 12 different genomic regions (**Supplementary Table S1**) in areas with known resistance-causing SNPs. All primers were tested on *E. coli-*K12 wild-type genomic DNA, and the PCR products were verified by 1% agarose gel electrophoresis with GelRed (Biotium, Freemont, CA, United States).

### Amplicon Library Preparation and Sequencing

All samples were amplified using the primers listed in **Supplementary Table S1** according to the descriptions in the above section for each drug condition. Each PCR reaction was performed in 0.2-mL sterile PCR tubes that contained 10-μL Phusion Flash High-Fidelity PCR Master Mix (Thermo Fisher Scientific, United States), 0.5 μM forward primer, 0.5 μM reverse primer, 1 μL DNA template and H_2_0 to a total reaction volume of 20 μL. The PCR amplification consisted of an initial denaturation at 98°C for 30 s, 30 cycles consisting of 98°C for 10 s denaturation, 65°C for 10 s annealing, 72°C for 15 s elongation, 72°C for 60 s for final extension, and subsequent holding at 4°C. To validate correct amplification, gel electrophoresis was performed with 1% agarose gel stained with ethidium bromide. After verification, all the different locus amplicons that belonged to the same drug condition, lineage and time point were pooled together. The PCR products were cleaned with an Agencourt AMPure XP PCR purification system according to the manufacturer’s online protocol (Beckman Coulter 2013). The barcoding of the amplicons was achieved via a second PCR amplification that was performed in 0.2-mL sterile PCR tubes that contained 25 μL Phusion Flash High-Fidelity PCR Master Mix (Thermo Fisher Scientific), 5 μL Nextera XT index 1 (N7XX; Illumina), 5 μM Nextera XT index 2 (S5XX; Illumina), 5 μL of the pooled amplicons and H_2_0 to a total reaction volume of 50 μL. The PCR amplification used an initial denaturation of 98°C for 30 s, 8 cycles consisting of 98°C for 10 s denaturation, 65°C for 10 s annealing, 72°C for 15 s elongation, 72°C for 60 s for final extension and subsequent holding at 4°C. The barcoded amplicons were cleaned according to the instructions of Agencourt AMPure XP PCR purification systems (Beckman Coulter 2013). The barcoded amplicons were measured for their DNA concentrations with Qubit dsDNA HS Assay kits on a Qubit Fluorometer (Thermo Fisher Scientific Inc. 2015) and average fragment size with Agilent DNA 1000 kits on an Agilent 2100 bioanalyzer instrument (Agilent Technologies 2013). Next, the barcoded amplicons were pooled based on fragment size and DNA concentration value to form a sequencing library. The sequencing library was sequenced on the MiSeq sequencing platform (Illumina, United States).

### Sequence Analysis

All raw sequencing reads from our amplicon sequencing have been deposited in the Sequence Read Archive (SRA) under the BioProject accession number PRJNA328094. The sequencing reads were mapped to the reference genome (GenBank accession NC_000913) using the CLC genomics workbench (Qiagen). These files were exported and used for coverage analysis. The CLC genomic workbench (Qiagen) and the statistical program R (R Core Team, 2014) were used to verify the sequencing coverage mapping read files. Coverage plots were generated, and any amplicons that were below our set threshold of 1000 read coverage were re-done until our threshold was achieved to ensure good quality data (**Supplementary Figures S1–S5**). To detect variants, all mappings were analyzed with the basic variant detection calling function of the CLC Genomic Workbench (Qiagen). The files were further analyzed using the program R (R Core Team, 2014). Our detected variants are listed in **Supplementary Table S2**.

### Recombineering for Direct Mutagenesis

For the single mutant variants, the ancestral wild-type *E. coli* K-12 MG1655 was transformed with the transient mutator plasmid PMA7sacB (GenBank accession KT285941) (Lennen et al., 2016). For each single variant mutation, two recombineering cycling rounds were performed with *E. coli* K-12 MG1655 harboring the PMA7sacB plasmid according to the MAGE cycling with the single-stranded oligonucleotides (ss-oligos) with verification primers (**Supplementary Table S3**) and plasmid curing described by Lennen et al. (2016). Direct mutagenesis verification was confirmed by Sanger sequencing performed with Eurofins Mix2Seq kits according to the manufacturer’s instructions (Eurofins Genomics, Esbersberg, Germany). After verification, the double-mutant variants were created by taking one of the single-mutant variants from the observed double-mutant pair and transforming it with the PMA7sacB plasmid. Recombineering with the other mutant variant ss-oligo was performed in two rounds as previously described. Plasmid curing was also performed.

### Characterization of the Recombineered Mutation Variant Strains

The relative growth rate was used as an indicator of fitness and was assessed for all the recombineered strains and wild type. To measure relative growth fitness, overnight cultures in LB medium were diluted to 1 × 10^6^ CFU/mL and then added to 96-well microtiter plates with a medium negative control. All procedures were performed in eight biological replicates with two technical replicates to allow for the calculation of the relative growth rate. The growth of the samples was done at 37°C with shaking for 10 s and kinetic measurements on ELx808 absorbance microplate reader with Gen5 software (BioTek Instruments Inc., Winooski, VT, United States). Optical density measurements were taken at 630 nm (OD_630_) every 10 min. Exponential growth was defined by OD_630_ values between 0.02 and 0.1. To calculate relative growth, R statistical software was used to find the doubling time (Td) of each of the strains and the standard deviation (Td SD) and then to calculate relative growth rate, and the recombineered strain Td value was divided by the wild-type Td value. Selective advantage was measured by performing IC90 determination assays with the antibiotic drug condition in which the mutations were observed (R Core Team, 2014). IC90 determination was performed in 96-well microtiter plates with a two-fold drug gradient that consisted of 10 drug concentrations that were prepared in LB broth; each well had a volume of 100 μL and was inoculated with 1 μL bacterial culture to provide each well with an initial inoculum of 1 × 10 CFU/mL. Each plate had eight biological replicates. The plates were incubated for 18 h at 37°C, and the OD_600_ values were read on a Synergy H1 plate reader (BioTek Instruments Inc., Winooski, VT, United States). The data were analyzed with Prism and IC90 values were calculated with point-to-point curve analysis Prism 6 (GraphPad Software Inc.).

To calculate the doubling time of each of the bacterial strains, overnight cultures in LB medium were diluted to 1 × 10^6^ CFU/mL and then added to 96-well microtiter plates to which a two-fold serial dilution of antibiotics was added with a medium negative control; all were performed with four biological replicates. Growth of the samples was performed at 37°C with shaking for 10 s, and kinetic measurements performed on ELx808 absorbance microplate reader with Gen5 software (BioTek Instruments Inc., Winooski, VT, United States). Optical density measurements were taken at 630 nm (OD_630_) every 10 min. Exponential growth was defined as OD_630_ values of between 0.02 and 0.1. To calculate relative growth, R statistical software was used to find the doubling time (Td) of each of the strains, as well as the standard deviation (Td SD) (R Core Team, 2014). Plots were generated with prism 6 (GraphPad Inc.).

## Results

### Selection of Loci for Population Sequencing

We specifically targeted putative resistance loci that were identified by Munck et al. (2014) through the whole-genome sequencing of evolved isolates. As a template, we used DNA isolated from the evolving populations that were sampled every 48 h during the experiment. The following genomic loci used for each drug condition based on previously end-point sequenced mutant alleles were: *cpxA*, *fusA*, and *sbmA* (AMK); *marR* and *rob* (CHL); *acrR*, *gyrA*, and *soxR* (CIP); *cpxA* and *marR* (AMK + CHL); and *acrR*, *marR*, *gyrA*, and *soxR* (CHL + CIP). These genes are implicated in either multi-drug resistance or specific-drug resistance. The genes implicated in multi-drug resistance include global regulators that induce the up-regulation of acrAB-TolC multi-drug efflux systems, i.e., *acrR*, *marR*, *rob*, and *soxR* (Chubiz et al., 2012). Mutations in these global regulators often have generalized cross-resistance effects across multiple antibiotic classes. By contrast, the genes implicated in specific-drug resistance are *gyrA* mutations (Fu et al., 2013) that confer CIP resistance and mutations of *cpxA* (Girgis et al., 2009), *fusA* (Johanson and Hughes, 1994), and *sbmA* (Chubiz et al., 2012; Puckett et al., 2012) that confer AMK resistance.

### Allele Dynamics Differ between Single-Drug and Drug-Combination Evolved Populations

Our sample collection consisted of 90 samples obtained from 15 bacterial populations (triplicate lineages of five drug conditions). Three of these drug conditions involved single drugs, i.e., AMK, CHL, and CIP, and the remaining two drug conditions involved the drug combinations of AMK–CIP and CHL–CIP. The triplicate parallel lineages were assigned letters from A–C and were sampled at six time points (days 2, 4, 6, 8, 10, and 12; **Figure 1A**). We extracted genomic DNA from these samples, amplified the loci of interest, verified the products by gel electrophoresis, pooled the amplicons comprising targeted alleles based on drug condition and time point, barcoded the amplicons with indices, and pooled these together to form a sequencing library. The library was sequenced using the MiSeq sequencing platform to establish the frequency of mutants in each of the selected loci (see Materials and Methods and **Figure 1B**). A minimum coverage of 1000 sequencing reads per amplicon was required before further data processing (**Supplementary Figures S1–S5**).

**FIGURE 2:**
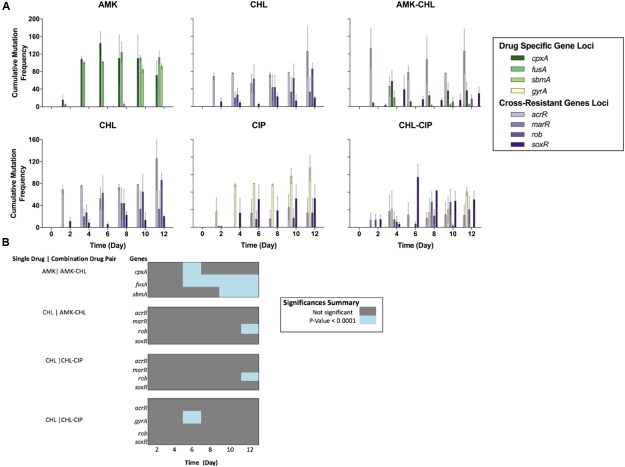
**Comparison of the cumulative mutational frequencies of the gene loci for each drug condition over the deep-sequencing time points (A)** The cumulative mutation frequencies calculated for each time point after the removal of all mutations below 5%; from left to right, AMK (showing the *cpxA*, *fusA*, and *sbmA* gene loci), CHL (showing the *acrR*, *marR*, *rob*, and *soxR* gene loci) and AMK-CHL (showing the *acrR*, *cpxA*, *fusA*, *marR*, *rob*, *sbmA*, and *soxR* gene loci), from left to right, CHL (showing the *acrR*, *marR*, *rob*, and *soxR* gene loci), CIP (showing the *acrR*, *gyrA*, *rob*, and *soxR* gene loci) and CHL-CIP (showing the *acrR*, *gyrA*, *marR*, *rob*, and *soxR* gene loci). **(B)** Heat-map illustrating the differences in the cumulative mutational frequencies of the gene loci of interest and the drug combinations compared with the single-drug counterparts at each time point based on a Holm-Sidak multiple comparison two-way ANOVA (all of the dark gray points are not significant, and all of the blue points are significant with *P*-values below 0.0001).

From this data, we compared the allele dynamics of the single-drug evolved populations with those of the combination drug-evolved populations. It is assumed that antibiotic combinations can suppress antibiotic resistance evolution compared with drug monotherapy. However, we have previously shown that this is not always the case; rather, only combinations with collateral sensitivity interactions seem to suppress drug evolution (Munck et al., 2014). We performed a Holm–Sidak multiple comparison two-way ANOVA statistical test to compare the cumulative frequency of mutations for the sequenced loci between the different drug conditions (**Figure 2**). We found spurious differences between the single- and combination-drug conditions in cumulative frequency of mutations for specific loci. However, two loci exhibited consecutive significant differences over two or more time points. These were the *fusA* gene and the *sbmA* gene in which mutations were significantly less frequent in the AMK–CHL drug-evolved populations compared with the AMK drug-evolved populations (*P*-value ≤ 0.001, Holm–Sidak multiple comparison two-way ANOVA test; **Figure 2B**). Similar results were observed in the work of Munck et al. (2014) and likely results from collateral sensitivity to CHL caused by these mutations (Macvanin et al., 2005; de Cristobal et al., 2008).

### Populations Exhibit Complex Allele Dynamics during Antibiotic Resistance Evolution

To focus on important alleles within our populations, we selected all alleles that appeared in at least one time point with a population frequency ≥ 30% (**Supplementary Figure S6**). We observed five mutations that occurred in parallel evolution events in different adapted populations (AMK: *cpxA* Trp184Arg, *fusA* Pro610Gln; AMK–CHL: *acrR* Lys55Glu; CIP and CHL–CIP: *gyrA* Ser83del; CHL–CIP: *soxR* Arg20Cys). Of these mutations, two have not previously been reported in the literature, namely, *acrR* Lys55Glu in AMK–CHL populations and *cpxA* Trp184Arg in AMK populations. We speculate that the *acrR* Lys55Glu mutation confers resistance to the CHL component in the AMK–CHL populations via the up-regulation of the acrAB-TolC multi-drug efflux pump, whereas *cpxA* Trp184Arg confers AMK resistance by modifying the membrane stress response.

The most prevalent mutation in the evolved bacterial populations was *gyrA* Ser83del, which was observed in three CHL-CIP and two CIP populations at several time points. The *gyrA* gene often mutates at amino acid position 83; however, it has only been reported once that the mutation was due to a deletion of the codon (Jaktaji and Mohiti, 2010), as seen within our data. We observed a high prevalence of this mutation, especially in the CHL-CIP lineage A; on day 12, this mutation was observed at a frequency of 100% in the population. However, this mutant allele was not detected by Munck et al. (2014) possibly due to the use of whole-genome sequencing of a representative isolate from the population that was performed on day 14. To support our findings, we have provided a picture of our population result (**Supplementary Figure S7**) and an additional Sanger sequencing result of the population to re-confirm our findings (**Supplementary Figure S8**).

**FIGURE 3:**
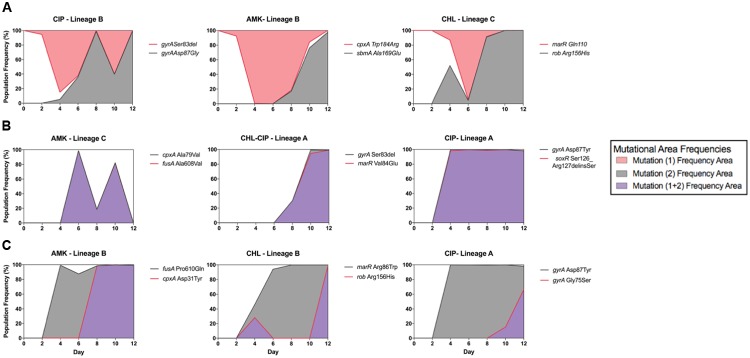
**Epistatic mutation interaction observation of the whole bacterial population.** Epistatic mutation interaction observation of the whole bacterial population with the three types of mutational interactions shown with three examples each of **(A)** all interfering allelic dynamics; the mutational areas form different fractions of the population shown in either red fill or gray fill, from left to right CIP-Lineage B (with *gyrA* Asp87Gly in gray line and fill and *gyrA* Ser83del in red line), AMK-Lineage B (with *sbmA* Ala169Glu in gray line and *cpxA* Trp184Arg in red line) and CHL-Lineage C (with *rob* Arg156His in gray and *marR* Gln110^∗^ in red line). **(B)** Super-imposing allelic dynamics in which both mutations are present at the same time. The mutational frequency area is in purple fill; in this situation, it is assumed that both mutations are co-existing in the bacterial cells, from left to right, AMK-Lineage C (with *cpxA* Ala79Val in gray line and *fusA* Ala608Val in red line), CHL–CIP Linage A (with *gyrA* Ser83del in gray line and *marR* Val84Glu in red line) and CIP Lineage A (with *gyrA* Ser83del in gray line and *soxR* Ser126_Arg127delinsSer in red line). **(C)** Population fixation allele dynamics in which the mutations that increase the frequency area are shown in gray fill. The second mutation arises in the background of the first; therefore, we assume that both are present within the bacterial cells; for this situation, purple frequency area fill was used, from left to right AMK-Lineage B (with *fusA* Pro610Gln in gray line and *cpxA* Asp31Tyr in red line), CHL-Lineage B (with *marR* Arg86Trp in gray line and *rob* Arg156His in red line) and CIP-Lineage A (with *gyrA* Asp87Tyr in gray line and *gyrA* Gly75Ser in red line).

Within our data, we observed three distinct allele dynamics (**Figure 3A**), which we termed as follows: interfering allele dynamics in which the mutant alleles have opposing dynamic trajectories (**Figure 3A**); super-imposing allele dynamics in which the mutant alleles have the same frequency trajectories (**Figure 3B**); and fixation allele dynamics in which one allele fixes in the population and provides the genetic background in which new alleles appear (**Figure 3C**).

We observed interfering allele dynamics in the following three populations: CIP lineage B, AMK lineage B, and CHL Lineage C. We assume that clonal interference is the most likely cause of our observations, but in some cases, it could be due to mutational incompatibility. For instance, we were able to demonstrate direct antagonistic interactions between the two mutations in the CIP lineage B because both mutations were captured within a single read. We observed that the individual reads from this lineage either had an amino acid change in *gyrA* at position Ser83del or Asp87Gly for the time points from 4 to 12. However, further experimentation is required to deduce which effect is responsible for our other observations of interfering allele dynamics.

We observed super-imposing allelic dynamics in the following three populations: AMK lineage C, CHL–CIP lineage A, and CIP lineage A. For all three populations, the two mutant alleles exhibited similar population frequencies over multiple time points (**Figure 3C**). Such fixation patterns likely result from the quick succession of two separate mutations within a cell. Because our population sequencing was performed only every 48 h, we likely were unable to resolve the successive occurrence of these mutations. These observations could be the result of two possible effects. The first is the hitchhiking effect in which a neutral mutant allele co-occurs with a beneficial mutant allele and, due to the selective advantage provided by the beneficial allele, the neutral mutation is therefore irreversible (Taddei et al., 1997; Tenaillon et al., 1999). The second is that both mutations contribute to the selected phenotype (Blount et al., 2008; Fogle et al., 2008; Wong and Seguin, 2015). We are not able to make a distinction between these two scenarios based on our allele frequency data alone.

For the fixation allele dynamics, we observed one allele that fixes at 100% in the population followed by the establishment of a subsequent allele. This pattern was observed in the following three populations: AMK lineage B, CHL lineage B, and CIP Lineage A.

In summary, we observed interfering allele dynamics in five populations, superimposing allele dynamics in seven populations interactions and fixation allele dynamics in eight populations (**Table 2**). This highlights that diverse population dynamic patterns can be observed during laboratory antibiotic resistance evolution.

**Table 2:** Observed allelic dynamics.

Dynamics	Drug condition	Lineage	Mutation(s)
Interfering (5)	AMK	A	*cpxA* Asp31Tyr + *fusA* Pro610Gln/*fusA* Pro610Thr
	AMK	B	*cpxA* Trp184Arg/*sbmA* Ala169Glu
	AMK	C	*cpxA* Ala79Val + *fusA* Ala608Gln/*fusA* Thr674Ala
	CHL	C	*marR* Gln110^∗^/*rob* Arg156His
	CIP	B	*gyrA* Asp87Gly/*gyrA* Ser83del
Super-imposing (7)	AMK	A	*cpxA* Asp31Tyr + *fusA* Pro610Gln
	AMK	B	*cpxA* Trp184Arg + *fusA* Ala608Gln
	AMK	C	*cpxA* Ala79Val + *fusA* Ala608Val
	CHL	B	*acrR* Ala156Ser + *rob* Arg156His
	CIP	A	*gyrA* Asp87Tyr + *soxR* Ser126_127delinsSer
	AMK–CHL	B	*acrR* Gln183Lys + *acrR* Met175Arg
	CHL–CIP	A	*gyrA* Ser83del + *marR* Val84Glu
Population Fixation (8)	AMK	A	*fusA* Pro610Thr
	AMK	A	*sbmA* Asp194Asn
	AMK	B	*cpxA* Trp184Arg + *fusA* Pro610Gln
	AMK	C	*cpxA* Trp184Arg
	CIP	A	gyrA Asp87Tyr + soxR Ser126_Arg127delinsSer
	CIP	C	*acrR* Ser25^∗^
	CIP	C	*gyrA* Gly81Cys
	CIP	C	*soxR* Arg127_Ser128delinsArg

*^∗^Non-sense mutation.*

### Recombineering Permits the Validation of Interactions between Genotypes

**FIGURE 4:**
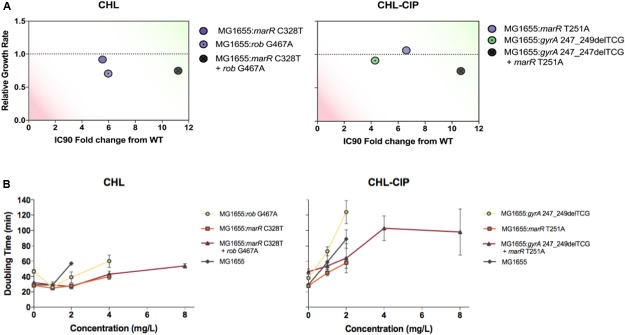
**Evaluation of the strains based on the relative growth rates and IC90 fold changes relative to the ancestral wild-type (green on the plots represents a relative growth rate ≥ 1 and a IC90 fold change from the wild type above ≥ 10, and red represents a relative growth rate ≤ 0.5 and a IC90 fold change from wild type above ≤ 2), and the doubling times at varying antibiotic concentrations (A).** Strains were recombineered to assess the clonal interference mutation interactions between genotypes based on mutations found in the CHL-Lineage C (the strains are labeled accordingly on the right-hand side). Strains were recombineered to access the super-imposing establishment mutation interactions between genotypes based on the mutation found in the CHL–CIP Linage A (the strains are labeled accordingly on the right-hand-side) **(B)**. The doubling times at various chloramphenicol concentrations were plotted for all recombineered strains and the ancestral wild type based on the mutation found in CHL-Lineage C (the strains are labeled accordingly on the right-hand side). The doubling times at various chloramphenicol + ciprofloxacin concentrations were plotted for all recombineered strains and the ancestral wild type based on the mutation found in the CHL–CIP Linage A (the strains are labeled accordingly on the right-hand side).

To follow up on our observations, we used recombineering to engineer strains with our observed mutant alleles. This process was performed with a subset of detected mutations that exhibited superimposing establishment or interfering allele dynamics. By measuring the relative growth rates and antibiotic tolerance of the isogenic mutants compared with those of the ancestral wild type, we were able to deduce the basis of some allele dynamics (**Figure 4**).

In the CHL lineage C population, we observed interfering allele dynamics. In particular, we observed that the *rob* G467A allele became dominant in the population and that the *marR* C328T was lost from the population from day 6 onward (**Figure 3A**). Therefore, we constructed three bacterial strains: MG1655: *rob* G467A, MG1655: *marR* C328T, and MG1655: *marR* C328T+*rob* G467A, and tested the relative growth rate and CHL IC90 for each strain relative to the wild type (**Figure 4A**). We find that the *rob* G467A strain was selected due to its increased antibiotic resistance compared with the *marR* C328T strain (**Figure 4A**). The *rob* G467A strain exhibited a 6-fold increase in the IC90 relative to the ancestral wild type, whereas the *marR* C328T strain exhibited a 5.5-fold increase. Even though this difference in IC90 values is small it suggests that the observed fixation patterns in the CHL lineage C population resulted from clonal interference and not mutational incompatibility. To validate this assumption, we constructed the double mutant *marR* C328T+*rob* G467A; this strain was viable and exhibited CHL IC90 11-fold higher than the ancestral wild type. The double mutant did exhibit a reduced growth rate in absence of antibiotics compared to the single mutants and the ancestral wild type. However, when quantifying the growth rate of the strains in the presence of different CHL concentrations we observed that the double mutant was able to sustain growth up to concentrations of 8 mg/L CHL (doubling time of 54 min), whereas both single-mutant allele strains could only sustain growth in CHL concentrations up to 2 mg/L (the doubling time for *marR* C328T and *rob* G467A was 40 and 60 min, respectively). Finally, the ancestral wild type could sustain growth in CHL concentrations up to 2 mg/L with a doubling time of 57 min (**Figure 4B**). Accordingly, the double mutant could have been selected for in the experiment at CHL concentrations above 2 mg/L.

For our super-imposing allele dynamics, we tested our observations in the CHL–CIP lineage A population in which *gyrA* 247_249delTCG and *marR* T251A appeared at the same frequency from day 8 (**Figure 3B**). These observed mutations likely complement each other since *gyrA* 247_249delTCG would confers target mediated resistance against CIP (Fu et al., 2013), and *marR* T251A induces up-regulation of the acrAB-TolC multi-drug efflux pump that causes collateral resistance to both CHL and CIP (Chubiz et al., 2012). Therefore, we recombineered three bacterial strains, i.e., MG1655: *gyrA* 247_249delTCG, MG1655: *marR* T251A, and MG1655: *gyrA* 247_249delTCG + *marR* T251A, and quantified the doubling time and CHL–CIP tolerance relative to the ancestral wild type (**Figure 4A**). We found that the double mutant exhibited strong selective advantage compared to the single mutants and the ancestral wild type in the presence of CHL–CIP. The IC90 of the double mutant was 11-fold higher than that of the ancestral wild type, and the corresponding values were 4.3-fold and 6.6-fold for the single mutants *gyrA* 247_249delTCG and *marR* T251A, respectively. The doubling time of the double mutant was 59% higher than the ancestral wild type when grown without antibiotic. Yet, in the presence of CHL–CIP the double mutant had a substantially lower doubling time than the single mutants and the ancestral wild type (**Figure 4**). The double mutant was able to sustain growth at concentrations of CHL–CIP up to 8.0116 mg/L (doubling time of 98 min). Both of the single-mutants could sustain growth at concentrations of CHL–CIP up to 2.004 mg/L (doubling time for *marR* T251A and *gyrA* 247_249del TCG was 58 and 124 min, respectively). The ancestral wild type sustained growth at CHL–CIP concentrations up to 2.004 mg/L (doubling time of 89 min). Based on these data it is obvious that if a double mutant was present in a population containing both single-mutants and wild type cells it would rapidly outcompete these other strains as antibiotic exposure increased.

## Discussion

There are many studies that report the genomic evolutionary trade-offs that occur during adaptive evolution and are supported by end-point whole-genome sequencing of single or multiple isolates (Lázár et al., 2013; Kim et al., 2014; Munck et al., 2014; Oz et al., 2014). Understanding these genomic trade-offs is essential for application of antibiotic collateral sensitivity treatments of drug cycling (where genomic evolution to one drug causes hyper-sensitivity to another drug) or combination treatments (where genomic evolution is constrained due to lack of genomic cross-resistance). These studies provide important genomic information but do not quantify the frequency of a given mutations in the whole population at the end of adaption or during adaptation. In this study, we present a method of time-resolved tracking of known genomic loci to evaluate the dynamics of putative resistance alleles.

We demonstrate the importance of tracking known genomic loci involved in antibiotic resistance to our drugs AMK, CHL, CIP, AMK–CHL, and CHL–CIP on a population level. We observe the reduced frequency of mutant alleles for *fusA* and *sbmA* in the AMK–CHL adapted populations compared to the AMK adapted populations. This is likely a result of the collateral sensitivity conferred by these mutant alleles toward CHL (Munck et al., 2014). However, we did observe *cpxA* mutant alleles develop in populations during adaptation toward AMK–CHL although at a reduced population frequency compared to AMK evolved populations.

We observed diverse allele dynamics in the adapting populations. These can be grouped into three categories: superimposing allele dynamics (Fogle et al., 2008); interfering allele dynamics (Gerrish and Lenski, 1998; de Visser and Rozen, 2006; Jerison and Desai, 2015); and fixation allele dynamics (Wilke, 2004; de Visser and Rozen, 2006; Good et al., 2012). All dynamic patterns where observed in this experiment highlighting the diversity of the evolutionary trajectories selected for during antibiotic resistance evolution.

Due to accessibility and reduced costs of next-generation sequencing, re-sequencing tools, such as amplicon population sequencing, are gaining popularity in the publication of other adaptive evolution studies (Ostman et al., 2012; Wong and Seguin, 2015; Feng et al., 2016) and the analyses of clinical and environmental samples (Johnning et al., 2015; Fischer et al., 2016; Greipel et al., 2016). While this method is powerful, it is limited in a number of ways. First, it requires *a priori* knowledge and selection of the loci to investigate. This selection likely leads to missing important mutants that occur outside the selected loci. Second, it is challenging to capture genomic duplication events such as occurs in adaptive evolution by the gene innovation–amplification–divergence model (Näsvall et al., 2012). Using our amplicon sequencing, we would assume that the diverged duplicated gene would confer resistance in our gene loci of interest, and in this instance, this assumption would be incorrect.

Nonetheless, we believe that these population wide loci specific approaches will play a critical role in *in vitro* adaptation evolution experiments and, with methodological modification, could extend to *in vivo* population sequencing studies. For example, with our sequencing method, we observed cross-resistance in bacterial clones within the CHL–CIP Lineage A population that was due to mutations in *gyrA* and *marR*, and a similar resistance mechanism has been described for fluoroquinolone resistance (Marcusson et al., 2009). To further evaluate this issue, we reconstructed an isogenic strain containing our *gyrA* and *marR* mutant, and we observed that this isogenic mutant strain was able to persist at four-fold greater CHL and CIP concentrations compared with its ancestral wild type. Although this strain did exhibit a fitness cost, if the double-mutant isogenic strain and the ancestral wild type were in an environment with a concentration of CHL + CIP above 1.002 mg/L, the double-mutant isogenic strain would outcompete the ancestral wild type. If this mutant was also able to undergo further evolution and develop fitness compensation mutations to restore fitness, then this strain would persist in environments with or without CHL and CIP (Andersson and Hughes, 2010).

These methodologies will be important in several situations, including the monitoring of chronic conditions that require long-term antibiotic treatment, i.e., chronic urinary tract infections (Blango et al., 2014; Nolan et al., 2015) and cystic fibrosis patients colonized with *Pseudomonas aeruginosa* (Folkesson et al., 2012) and *Staphylococcus aureus* infections (Vanderhelst et al., 2012).

## Author Contributions

Study conception and design: RH, CM, and MS; acquisition of the data: RH; analysis and interpretation of the data: RH and CM; drafting of the manuscript: RH; critical revision: RH, CM, and MS; and funding of the research: MS. All authors read and approved the final manuscript.

## Conflict of Interest Statement

The authors declare that the research was conducted in the absence of any commercial or financial relationships that could be construed as a potential conflict of interest.

## Supplementary Material

Figure S1 **Coverage plots for each investigatory amplicon for the AMK drug condition**.

Figure S2 **Coverage plots for each investigatory amplicon for the CHL drug condition**.

Figure S3 **Coverage plots for each investigatory amplicon for the CIP drug condition**.

Figure S4 **Coverage plots for each investigatory amplicon for the AMK–CHL drug condition**.

Figure S5 **Coverage plots for each investigatory amplicon for the CHL–CIP drug condition**.

Figure S6 **Significant variant tracking for each drug condition in which the mutations that appear at 30% or more at any given time point are plotted.** Mutations listed in black appear in all three lineages, those in dark gray appear in two lineages, and those in light gray appear only in one lineage. From left to right, AMK Lineage A [featuring the *cpxA* (dark green fill): Asp31Tyr (circle), Trp184Arg (square), *fusA* (mid-dark green fill): Pro610Gln (up-triangle), Pro610Thr (down-triangle); and *sbmA* (lightest green fill): asp194Asn (hexagon with dot) mutation], AMK Lineage B illustration of mutations *cpxA* (dark green fill): Asp31Tyr (circle), Trp184Arg (square); and *fusA* (mid-dark green fill): Pro610Gln (up-triangle), *sbmA* (lightest green fill): Asp194Glu (circle with dot). AMK Lineage C illustration of the *cpxA* (dark green fill): Asp31Tyr (diamond with dot), Trp184Arg (square); *fusA* (mid-dark green fill): Ala608Val, Pro610Gln (up-triangle), Pro610Thr (down-triangle), Thr647Ala (square with diagonal cross); *sbmA* (lightest green fill): Trp250^∗^ (diamond with cross). From left to right, CHL Lineage A [featuring the mutation *rob* (mid-dark purple): Gly245Arg (star)], CHL Lineage B [featuring the mutations *acrR* (light purple): Ala156Ser (circle with diagonal cross); *marR* (mid-light purple): Arg86Trp (square); and rob (mid-dark purple): Arg156His circle]. CHL Lineage C illustration of the mutations *acrR* (light purple): Arg209fs (hexagon); *marR* (mid-light purple): Arg86Trp (square), Gln110^∗^ (down-triangle); and *rob* (mid-dark purple): Arg156His (circle). From left to right, CIP Lineage A [featuring the mutations *gyrA* (mid-light green): asp87Tyr (circle), Gly75Ser (up-triangle), ser83del (square); soxR (dark purple): Ser126_Arg127delinSer (diamond with cross)]. CIP Lineage B illustration of the mutations *gyrA* (mid-light green): asp87Gly (diamond), ser83del (square); *rob* (mid-dark purple): and Arg156His (hexagon with diagonal cross). CIP Lineage C illustration of the mutations *acrR* (light purple): Ser25^∗^ (hexagon); *gyrA* (mid-light green): Gly81Cys (circle with diagonal cross), *soxR* (dark purple): Arg127_Ser128delinsArg (down-triangle). From left to right, AMK–CHL Lineage A [featuring the mutations *acrR* (light purple): Lys55Glu (circle); *cpxA* (dark green): Ala187Thr (square), Ile382_Thr383delinMetLeu (up-triangle), Ser175Cys (down-triangle); and *soxR* (dark purple): Ala18Glu (hexagon), glu150fs (square with diagonal cross)]. AMK–CHL Lineage B [featuring mutations; *acrR* (light purple): Asp157Asn (star), Cys205Phe (square), Lys55Glu circle and Met175Arg] and AMK–CHL Lineage C [featuring *acrR* (light purple): Ala156Ser (diamond) with Ala156Ser, Arg209fs (square with diagonal cross), Lys55Glu; *cpxA* (dark green): Ala187Thr and soxR (dark purple) Ala18Glu hexagon]. From left to right, CHL–CIP Lineage A [featuring the mutations gyrA (mid-light green): Phe96fs (circle), Ser83del (square); marR (mid-light purple): Cys51Ser (half-dark up-triangle), Val84Glu (down triangle); *soxR* (dark purple): Arg20Cys square] CHL–CIP Lineage B [the mutations *acrR* (light purple): Cys205delinsCysArg (circle), Thr5Ala (half dark circle); *gyrA* (mid-light green): Ser83del (square); *soxR* (dark purple): Arg20Cys (square), Ser126_Arg127delinsCys (diamond)] and CHL–CIP Lineage C [the mutations *acrR* (light purple): Cys205delinsCysArg (circle); *gyrA* (mid-light green): Ser83del (square); *soxR* (dark purple): Arg20Cys (square), Ser126_Arg127delinsSer (up-triangle)].

Figure S7 **Population verification of CHL-CIP Lineage A Day 12**.

Figure S8 **Sanger sequence verification of CHL-CIP Lineage A Day 12**.

TABLE S1 **Primers used for the generation of the amplicons**.

TABLE S2 **Variant detection table**.

TABLE S3 **Homologous recombineering oligos and verification primers**.
