# Supplementary figures and images for: Time-Resolved Tracking of Mutations Reveals Diverse Allele Dynamics during Escherichia coli Antimicrobial Adaptive Evolution to Single Drugs and Drug Pairs
Source: Front Microbiol. 2017 May 24;8:893. doi: 10.3389/fmicb.2017.00893 (PMC5442168; doi:10.3389/fmicb.2017.00893)

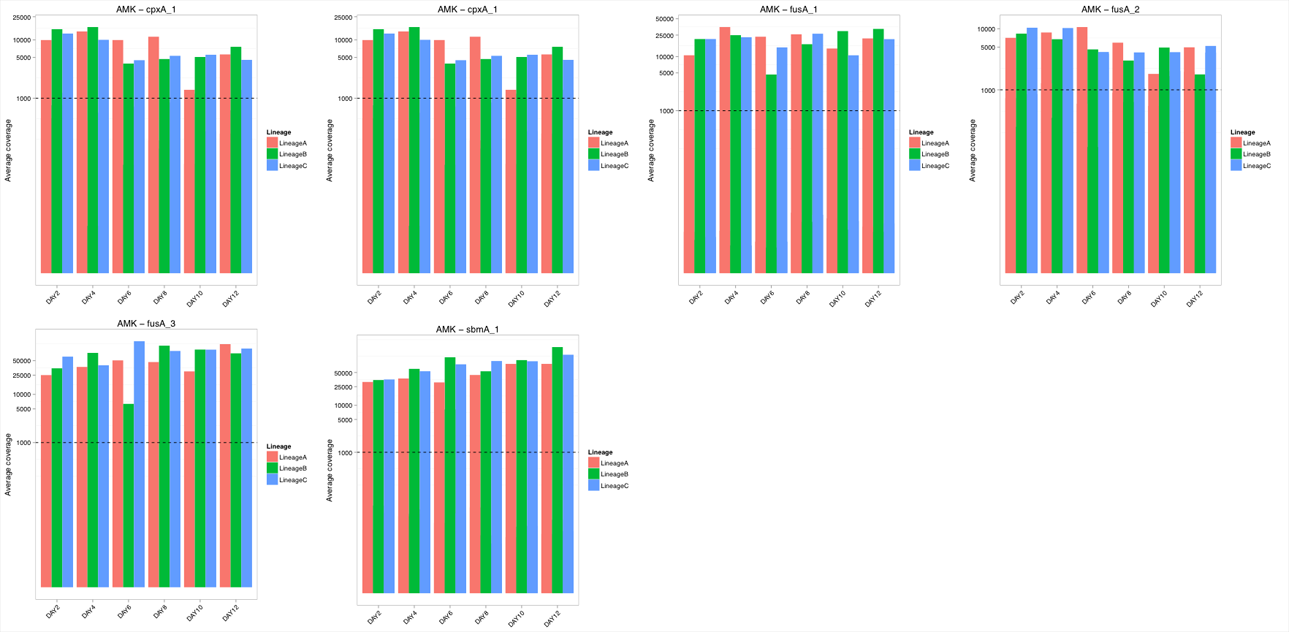

Supplement: Figure S1 — Coverage plots for each investigatory amplicon for the AMK drug condition. [file Image_1.TIFF]

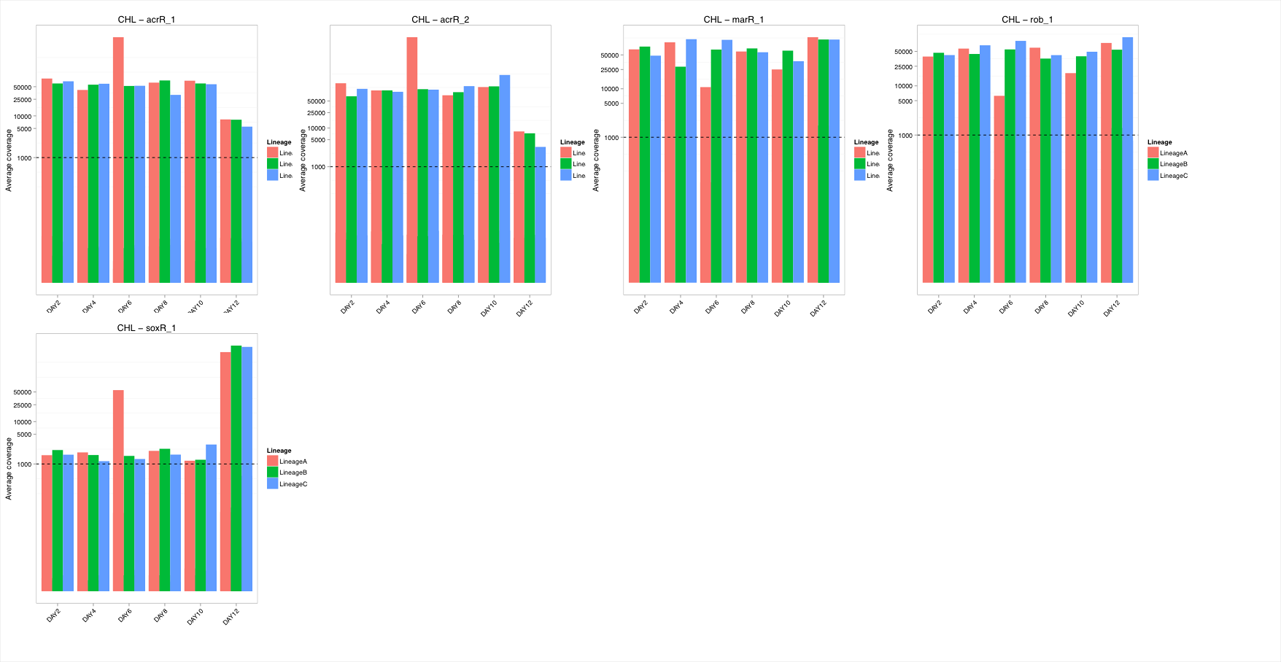

Supplement: Figure S2 — Coverage plots for each investigatory amplicon for the CHL drug condition. [file Image_2.TIFF]

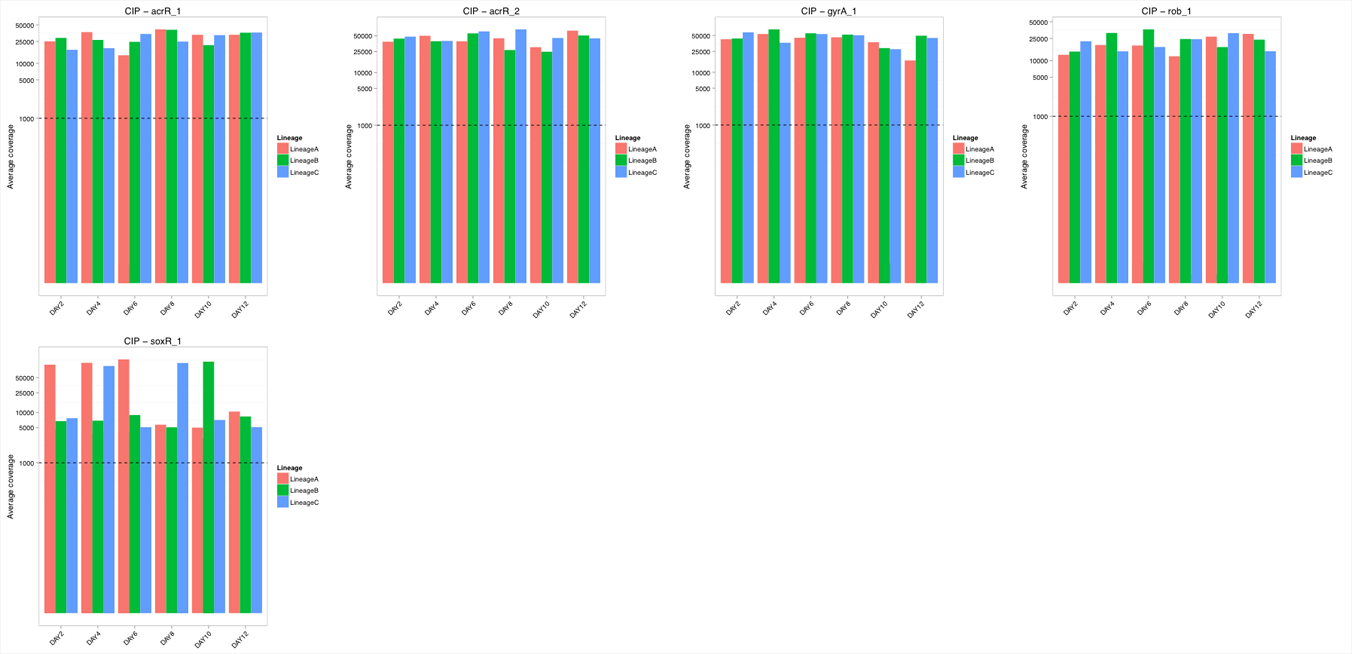

Supplement: Figure S3 — Coverage plots for each investigatory amplicon for the CIP drug condition. [file Image_3.TIFF]

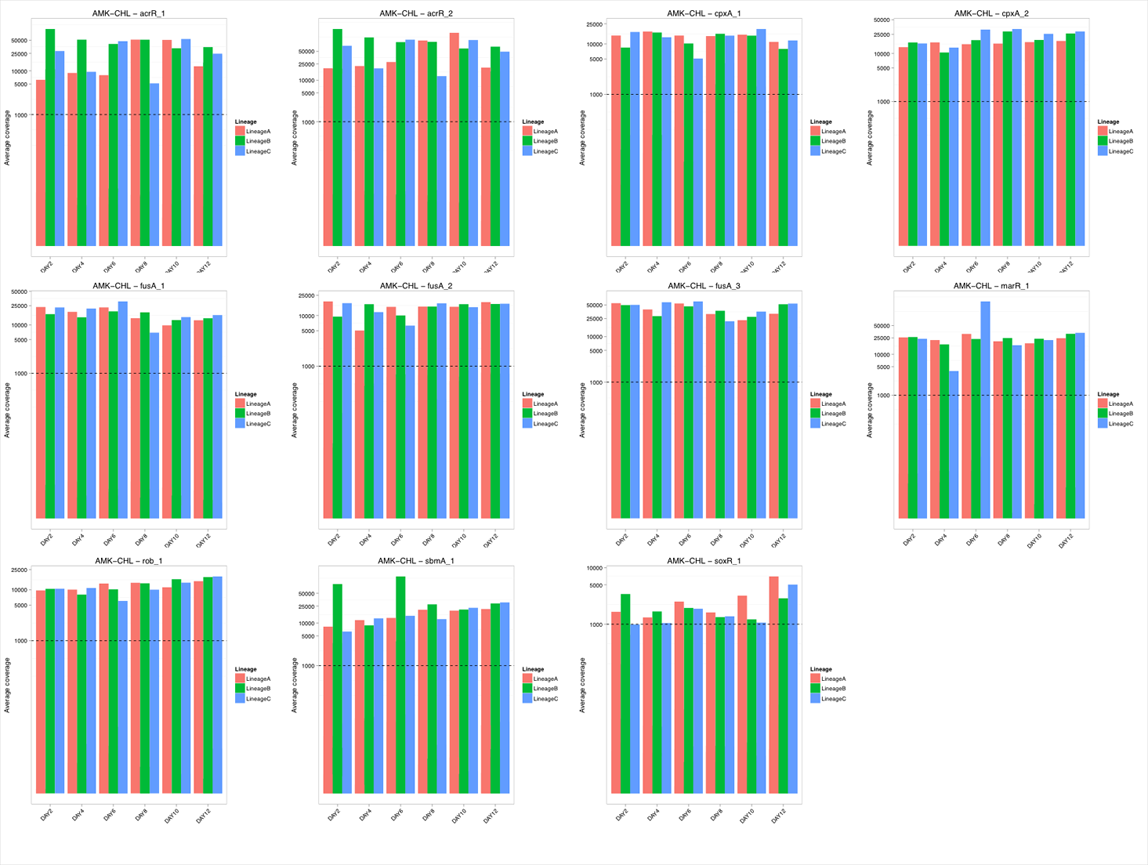

Supplement: Figure S4 — Coverage plots for each investigatory amplicon for the AMK–CHL drug condition. [file Image_4.TIFF]

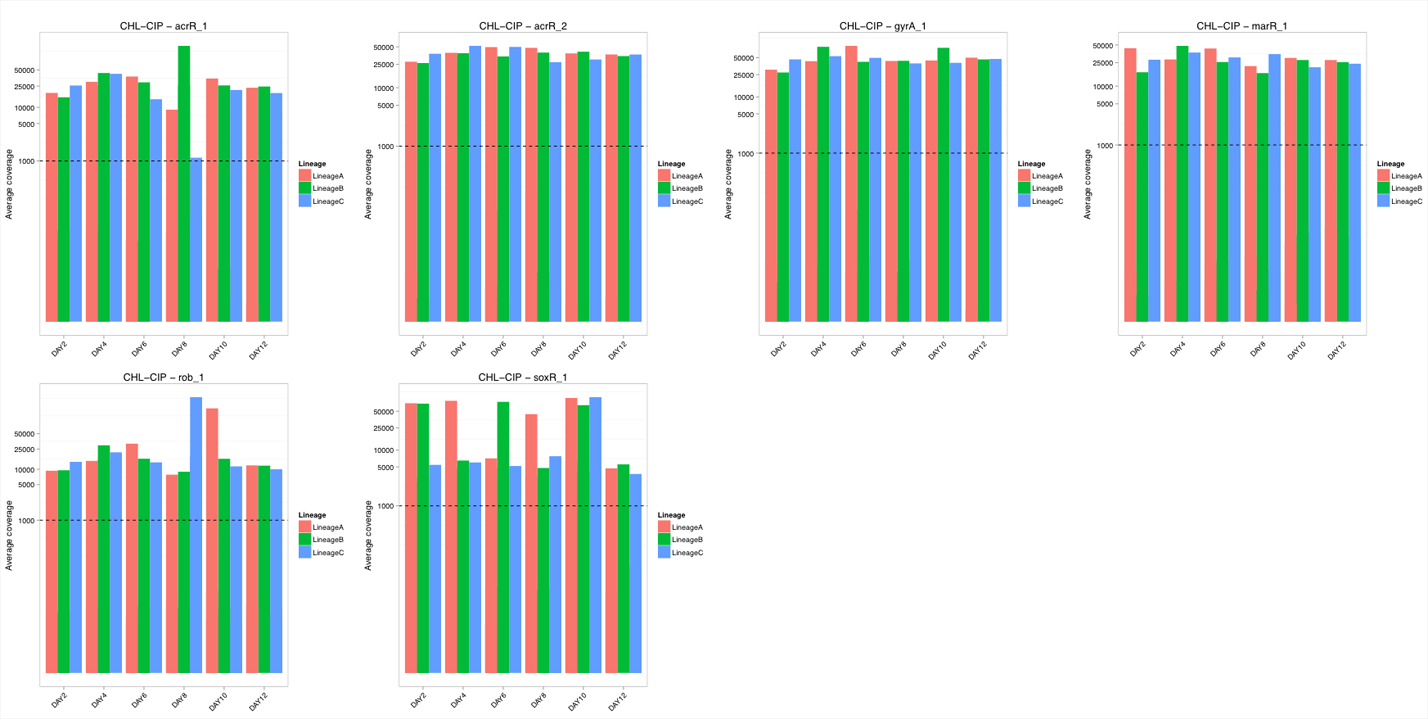

Supplement: Figure S5 — Coverage plots for each investigatory amplicon for the CHL–CIP drug condition. [file Image_5.TIFF]

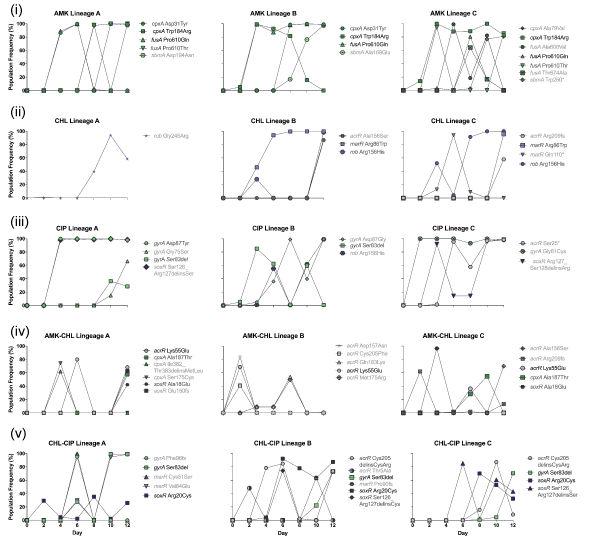

Supplement: Figure S6 — Significant variant tracking for each drug condition in which the mutations that appear at 30% or more at any given time point are plotted. Mutations listed in black appear in all three lineages, those in dark gray appear in two lineages, and those in light gray appear only in one lineage. From left to right, AMK Lineage A [featuring the cpxA (dark green fill): Asp31Tyr (circle), Trp184Arg (square), fusA (mid-dark green fill): Pro610Gln (up-triangle), Pro610Thr (down-triangle); and sbmA (lightest green fill): asp194Asn (hexagon with dot) mutation], AMK Lineage B illustration of mutations cpxA (dark green fill): Asp31Tyr (circle), Trp184Arg (square); and fusA (mid-dark green fill): Pro610Gln (up-triangle), sbmA (lightest green fill): Asp194Glu (circle with dot). AMK Lineage C illustration of the cpxA (dark green fill): Asp31Tyr (diamond with dot), Trp184Arg (square); fusA (mid-dark green fill): Ala608Val, Pro610Gln (up-triangle), Pro610Thr (down-triangle), Thr647Ala (square with diagonal cross); sbmA (lightest green fill): Trp250∗ (diamond with cross). From left to right, CHL Lineage A [featuring the mutation rob (mid-dark purple): Gly245Arg (star)], CHL Lineage B [featuring the mutations acrR (light purple): Ala156Ser (circle with diagonal cross); marR (mid-light purple): Arg86Trp (square); and rob (mid-dark purple): Arg156His circle]. CHL Lineage C illustration of the mutations acrR (light purple): Arg209fs (hexagon); marR (mid-light purple): Arg86Trp (square), Gln110∗ (down-triangle); and rob (mid-dark purple): Arg156His (circle). From left to right, CIP Lineage A [featuring the mutations gyrA (mid-light green): asp87Tyr (circle), Gly75Ser (up-triangle), ser83del (square); soxR (dark purple): Ser126_Arg127delinSer (diamond with cross)]. CIP Lineage B illustration of the mutations gyrA (mid-light green): asp87Gly (diamond), ser83del (square); rob (mid-dark purple): and Arg156His (hexagon with diagonal cross). CIP Lineage C illustration of the mutations acrR (light [file Image_6.TIF]

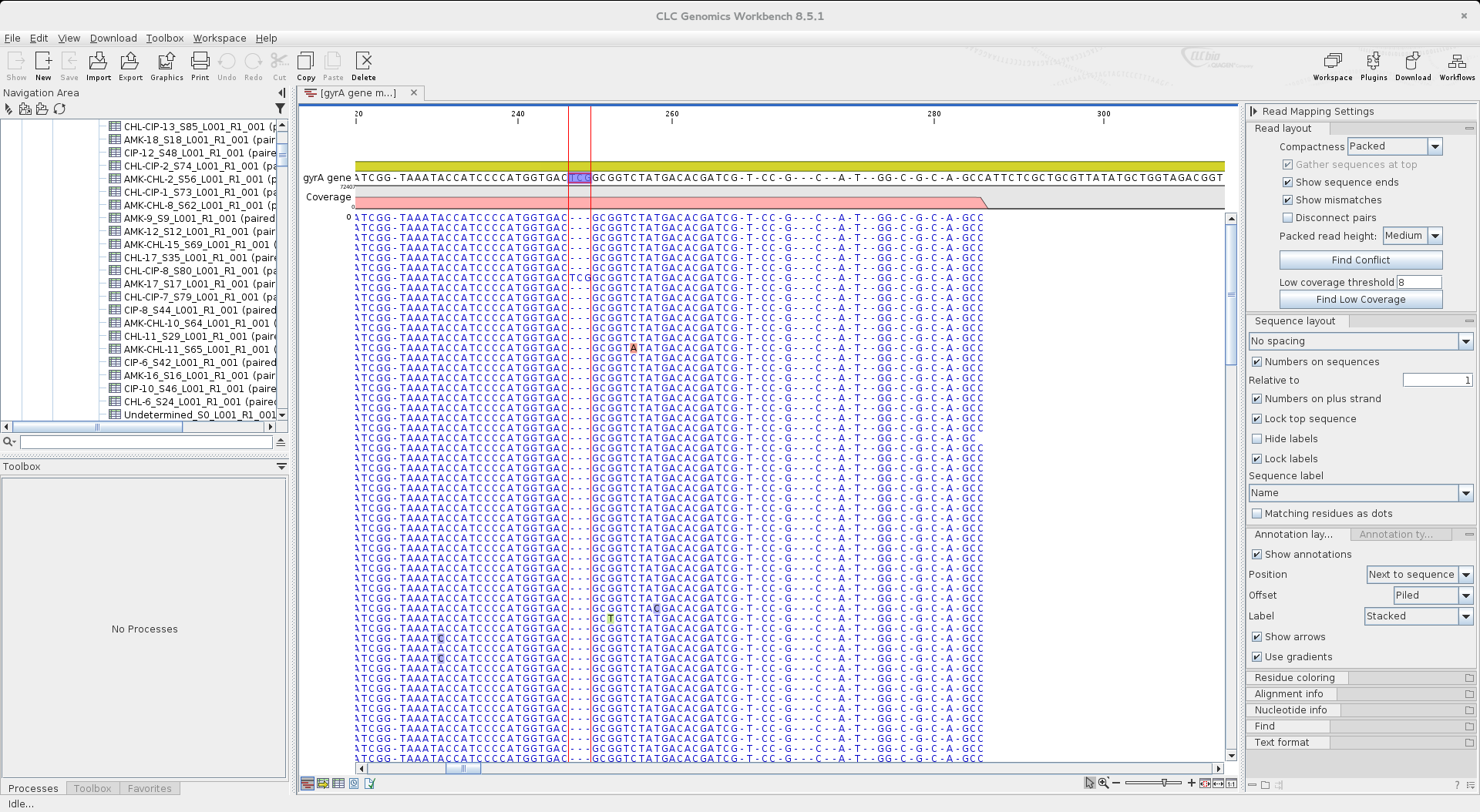

Supplement: Figure S7 — Population verification of CHL-CIP Lineage A Day 12. [file Image_7.png]

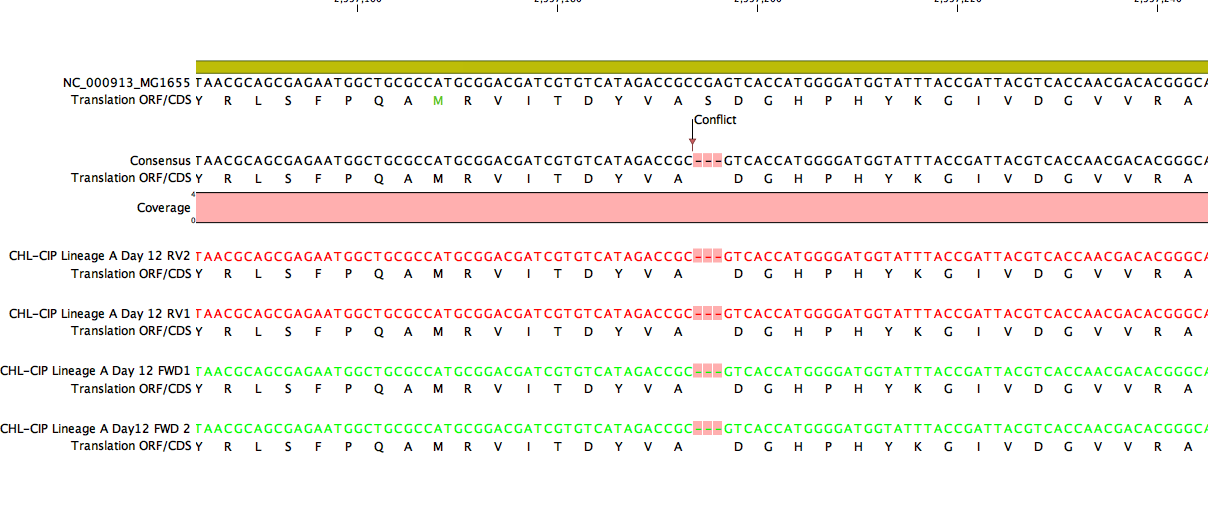

Supplement: Figure S8 — Sanger sequence verification of CHL-CIP Lineage A Day 12. [file Image_8.png]
